# Transgenic Expression of the *Helicobacter pylori* Virulence Factor CagA Promotes Apoptosis or Tumorigenesis through JNK Activation in *Drosophila*

**DOI:** 10.1371/journal.ppat.1002939

**Published:** 2012-10-18

**Authors:** Anica M. Wandler, Karen Guillemin

**Affiliations:** Institute of Molecular Biology, University of Oregon, Eugene, Oregon, United States of America; Stanford University, United States of Ameirca

## Abstract

Gastric cancer development is strongly correlated with infection by *Helicobacter pylori* possessing the effector protein CagA. Using a transgenic *Drosophila melanogaster* model, we show that CagA expression in the simple model epithelium of the larval wing imaginal disc causes dramatic tissue perturbations and apoptosis when CagA-expressing and non-expressing cells are juxtaposed. This cell death phenotype occurs through activation of JNK signaling and is enhanced by loss of the neoplastic tumor suppressors in CagA-expressing cells or loss of the TNF homolog Eiger in wild type neighboring cells. We further explored the effects of CagA-mediated JNK pathway activation on an epithelium in the context of oncogenic Ras activation, using a *Drosophila* model of metastasis. In this model, CagA expression in epithelial cells enhances the growth and invasion of tumors in a JNK-dependent manner. These data suggest a potential role for CagA-mediated JNK pathway activation in promoting gastric cancer progression.

## Author Summary

The gastric pathogen *Helicobacter pylori* infects an estimated 50% of the world's population and is a major risk factor for the development of gastric cancer. Strains of *H. pylori* that can inject the CagA effector protein into host cells are known to be more virulent, but the potential contributions of host genetics to pathogenesis are not well-understood. Using transgenic *Drosophila melanogaster*, we show that the genetic context of both the host cells in which CagA is expressed and their neighboring cells changes CagA's effects on epithelial tissue. When CagA is expressed in a subset of cells within an epithelium, it disrupts tissue integrity and induces apoptosis through activation of JNK signaling, a pathway that functions to remove aberrant cells from an epithelium. CagA's proapoptotic effects are inhibited by neoplastic tumor suppressor genes in CagA-expressing cells, and by the tumor necrosis factor homolog Eiger in neighboring cells. In contrast, when CagA is coexpressed with oncogenic Ras in a *Drosophila* model of metastasis, it enhances the growth and invasion of tumors in a JNK-dependent manner. Our study demonstrates how changes in host genetics can cooperate with activation of JNK signaling by the bacterial virulence factor CagA to promote tumorigenesis.

## Introduction

Infection with *Helicobacter pylori* is the strongest risk factor for the development of gastric carcinoma, which is the second most common cause of cancer-related death worldwide [1]. Although approximately half the world's population is infected with *H. pylori*, most of those individuals will develop simple gastritis and remain asymptomatic. However, 10–15% of infected subjects will develop duodenal ulcers and 1% will develop gastric adenocarcinoma [2]. This dramatic variability in clinical outcome of *H. pylori* infection is not well-understood, but likely results from the consequences of long-term interactions between the bacterium and its human host.

Specific bacterial and host genetic factors have been shown to affect *H. pylori* pathogenesis. Strains that possess the *cag* pathogenicity island (*cag* PAI), which encodes a type IV secretion system used to inject the CagA effector protein directly into gastric epithelial cells, are much more virulent [3]. Once inside host cells, CagA is tyrosine phosphorylated on conserved carboxyl terminal EPIYA motifs by Src family kinases. Variability in the number and composition of these phosphorylation motifs also correlates with differences in the carcinogenic potential of *H. pylori* strains [4]. Host genetic factors that can influence the progression and ultimate disease outcome of *H. pylori* pathogenesis include polymorphisms that enhance expression of certain cytokines [2], and genetic changes that occur during progression from normal mucosa to gastric carcinoma such as loss of tumor suppressors and activation of oncogenes [5]. Although development of a complex disease like gastric cancer requires the cooperation of many bacterial and host genetic factors, it is clear that the CagA effector protein is an important driver of disease progression.

CagA has been shown to interact with a multitude of host cell proteins belonging to several conserved signaling pathways [6], and these interactions are thought to promote carcinogenesis upon *H. pylori* infection. The majority of these interactions were discovered using cell culture models in which CagA expression can disrupt processes such as tight junction formation, motility and cytoskeleton dynamics. However, which interactions between CagA and host cell signaling pathways trigger the processes that lead to gastric cancer remains unclear [4]. Obtaining more specific information about the relative importance of CagA's interactions with host cell proteins will require investigation of their downstream effects on intact epithelial tissue.

In order to examine the effects of both bacterial and host genetic factors, our group has developed a system in which *Drosophila melanogaster* is used to model pathogenesis of the *H. pylori* virulence factor CagA [7]. There are several properties that make this model organism well-suited for studying the pathogenic effects of CagA expression. First, many canonical cell signaling pathways have been extensively characterized in *Drosophila* and show high conservation with the homologous pathways in humans. Also, genetic tools like the GAL4/UAS system allow expression of CagA in specific cells within an epithelium and examination of how CagA-expressing cells interact with neighboring wild type cells. Finally, we can easily manipulate host genes using resources generated by the rich *Drosophila* research community to assess potential effects on CagA-induced phenotypes. In addition, our model allows us to test whether CagA's interactions are phosphorylation-dependent through expression of a mutant form of CagA known as CagA^EPISA^, in which the EPIYA phosphorylation motifs have been deleted or mutated [8]. Use of this model has already provided insight into CagA's role in manipulating receptor tyrosine kinases, the Rho signaling pathway and epithelial junctions [7]–[10].

Epithelial polarity is one important feature of host cells known to be perturbed by CagA. Strains of *H. pylori* that encode CagA are exclusively able to cause localized disruption of apicobasal polarity in order to colonize a polarized monolayer of tissue culture cells [11]. CagA-positive strains of *H. pylori* have also been shown to cause apoptosis in both cultured gastric cancer cells and human gastric biopsies [12], [13], although the role of CagA-dependent apoptosis in *H. pylori* pathogenesis remains controversial. Loss of epithelial cell polarity has been shown to induce apoptotic cell death or promote tumorigenesis in different cellular and genetic contexts [14]. Cell death resulting from polarity disruption can trigger compensatory proliferation in order to replace lost cells, but this process can become tumorigenic in the presence of genetic alterations that block apoptosis [15]. This mechanism has been proposed to explain how the ability of CagA to disrupt cell polarity and induce apoptosis may be linked to its tumorigenic potential, but the host cell signaling pathways that could mediate these downstream effects have not been identified [16].

An important host signaling pathway that triggers apoptosis downstream of cell polarity disruption is the c-Jun NH_2_-terminal kinase (JNK) pathway. JNK is a stress-activated protein kinase with numerous upstream activators including cytokines, mitogens, osmotic stress, ultraviolet radiation and loss of cell polarity [17]. JNK-mediated apoptosis plays a role in several physiological processes including morphogenetic apoptosis and classical cell competition in which slow-growing cells are eliminated by their wild type neighbors. The JNK pathway also triggers apoptosis in response to a unique form of cell competition known as intrinsic tumor suppression where JNK activation performs a cell editing function by removing aberrant cells that arise within an epithelium, thus enhancing the resilience of epithelia to insult. Both expression of the tumor necrosis factor (TNF) homolog Eiger (Egr) and the presence of wild type cells within an epithelium are required for JNK pathway activation downstream of cell polarity disruption, and their absence can lead to tumor formation [18]. Furthermore, JNK signaling has been shown to switch from a proapoptotic to a progrowth role in the presence of oncogenic Ras [19]. These functions of the JNK pathway are well-established in *Drosophila*, and likely also relevant in mammals given the high conservation of this pathway throughout evolution [20].

Bacterial activation of JNK signaling has also demonstrated importance in enhancing epithelial robustness. During oral infection of *Drosophila* with the human pathogen *Pseudomonas aeruginosa*, the bacterium activates JNK signaling in the intestinal epithelium to trigger apoptosis and subsequent compensatory proliferation, thereby stimulating epithelial renewal. The same effect was not seen during infection with an avirulent strain of *P. aeruginosa* that does not secrete the virulence factor pyocyanin, suggesting a role for this effector protein in activating JNK signaling in response to damage induced by the bacterium [21]. Similar to the adult *Drosophila* intestine, the larval imaginal disc epithelia are particularly resistant to the effects of stress-induced apoptosis and can recover after losing over 50% of their cells during development to produce normal adult structures [22]. This inherent epithelial resilience makes the imaginal discs a relevant tissue in which to examine potential effects of JNK-dependent apoptosis mediated by a bacterial virulence factor.

In this study, we discovered a role for the CagA virulence factor in activating JNK signaling. We used transgenic *Drosophila* to express CagA in the developing wing imaginal disc, a simple polarized epithelial structure formed during larval stages of development. We found that CagA expression caused a distinct pattern of cell death in which apoptotic cells are basally extruded from the epithelium. In addition we showed that this apoptosis phenotype is enhanced by coexpression with Basket (Bsk), the *Drosophila* homolog of JNK, and suppressed by coexpression with a dominant-negative form of Bsk. From these results, we conclude that expression of CagA triggers JNK pathway activation which causes apoptosis in an intact epithelium. Furthermore, we used a *Drosophila* model of metastasis to show that CagA expression can enhance the growth and invasion of tumors generated by expression of activated Ras. This increase in tumorigenic capacity is suppressed by coexpression with dominant-negative Bsk, leading us to conclude that CagA promotes tumor growth and invasion through JNK pathway activation.

## Results

### CagA expression in the *Drosophila* wing causes apoptosis and epithelial disruption

**Figure 1:**
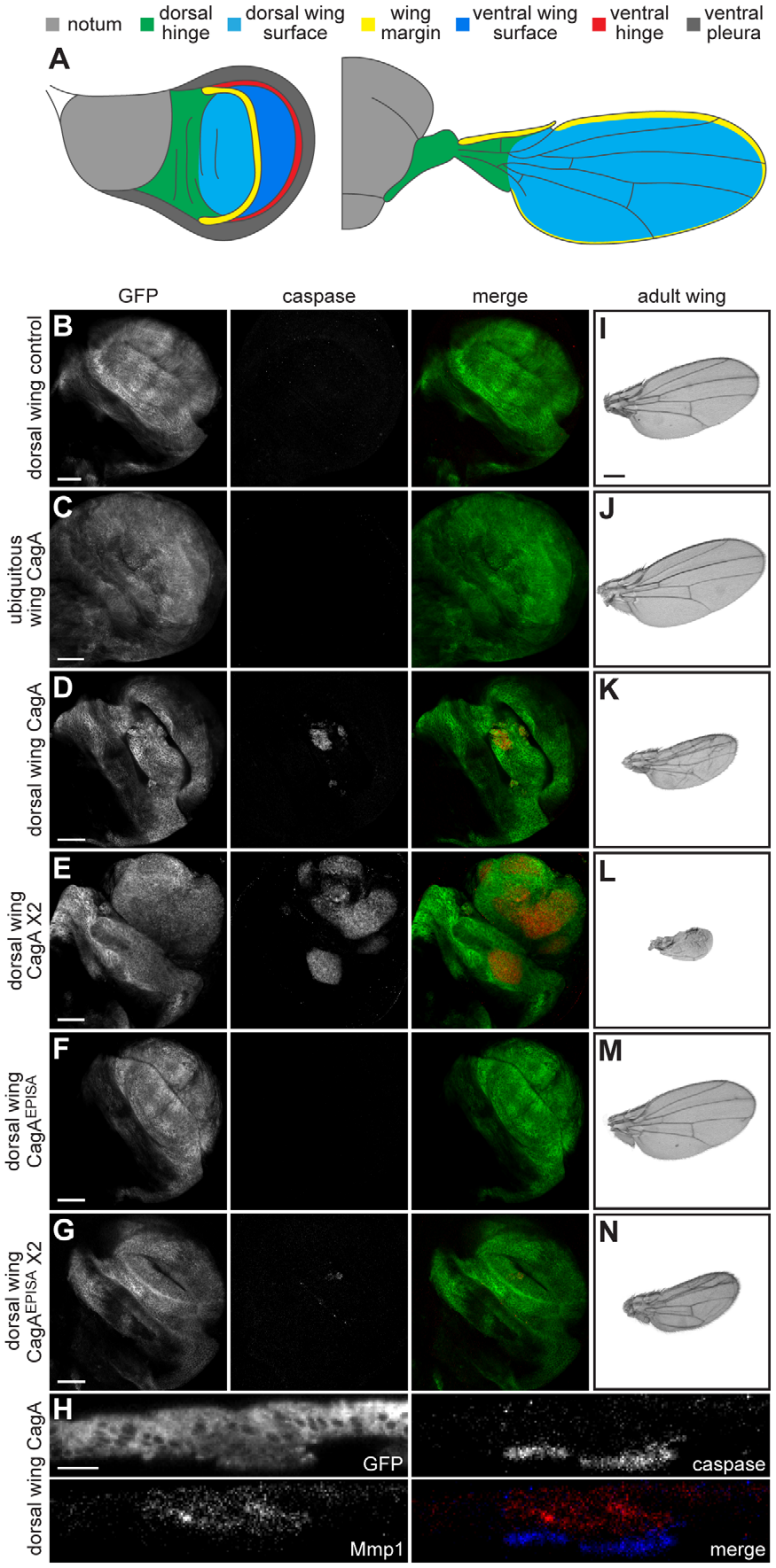
CagA expression causes apoptosis and epithelial disruption. (A) Schematic illustrating the fate of different domains within the wing imaginal disc. Each color-coded region of the larval structure on the left corresponds to the specified region of the adult wing on the right (modified from [24]). (B–G) Confocal cross sections of male third instar larval wing imaginal discs showing mGFP expression and stained with an antibody against active caspase-3 to mark apoptotic cells. A control wing disc epithelium expressing only mGFP with the bx-GAL4 dorsal wing driver (B) lacks apoptotic cells. Ubiquitous expression of CagA in the wing disc with the 765-GAL4 driver (C) does not cause apoptosis, while expressing CagA with bx-GAL4 (D) triggers formation of apoptotic clusters within the expression domain. Expressing two copies of CagA with bx-GAL4 (E) causes a dose-dependent enhancement of the apoptosis phenotype. Expressing CagA^EPISA^ with bx-GAL4 (F) does not cause a phenotype, while expressing two copies of CagA^EPISA^ (G) produces small apoptotic clusters. Scale bars, 50 µm. (H) XZ confocal plane of a male wing imaginal disc epithelium expressing mGFP and CagA with bx-GAL4 stained with antibodies against active caspase-3 to show basal extrusion of apoptotic cells and matrix metalloproteinase 1 (Mmp1) to show evidence of basement membrane breakdown. Scale bar, 20 µm. (I–N) Adult wing images from male flies of each indicated genotype. Neither expression of mGFP alone with bx-GAL4 (I) nor expression of CagA with 765-GAL4 (J) causes a phenotype in the adult wing. Dorsal wing expression of CagA with bx-GAL4 (K) disrupts epithelial integrity in a dose-dependent manner (L). Expressing CagA^EPISA^ with bx-GAL4 (M) does not cause an adult wing phenotype, while expressing two copies of CagA^EPISA^ (N) causes epithelial disruption. Scale bar, 500 µm.

In order to examine the effects of expressing the *H. pylori* effector protein CagA on an intact epithelium, we used the GAL4/UAS system to drive its expression in the wing imaginal disc. The *Drosophila* wing begins to form during early larval life when it exists as a primordial sac which contains both a simple columnar epithelium and the squamous epithelium of the peripodial membrane [23]. Cells within the wing imaginal disc proliferate extensively in larval stages followed by disc evagination during pupation, resulting in the adult wing structure. This developmental process is distinct from that of the eye imaginal disc used to model CagA pathogenesis previously [7]–[10], which undergoes systematic differentiation during larval stages. In addition, the fate of imaginal disc cells is specified early in development [24] which allowed us to express CagA in distinct regions of the wing disc (Figure 1A).

We expressed CagA with various GAL4 drivers specific to the wing (Figure S1A), and determined that both the level of CagA protein and the region in which it is expressed affect the resulting larval and adult wing phenotypes (Figure S1B–S1L). We focused our subsequent analysis on two different GAL4 drivers which express CagA either in a subset of wing cells or throughout the wing imaginal disc: beadex-GAL4 (bx-GAL4) is expressed specifically in cells of the columnar epithelium that give rise to the dorsal surface of the wing blade (Figure 1B), and 765-GAL4 is expressed ubiquitously throughout the wing. A membrane-localized GFP construct (mGFP) was used to visualize the expression domain. Expressing CagA with the 765-GAL4 ubiquitous wing driver did not cause any observable phenotype (Figure 1C). However, expressing CagA with the bx-GAL4 dorsal wing driver caused clusters of apoptotic cells to form near the center of the expression domain in wing imaginal discs from third instar larvae (Figure 1D). This phenotype was dose-dependent, since expressing two copies of CagA increased both the size and number of apoptotic clusters formed (Figure 1E). A similar phenotype has been shown to result from localized JNK pathway activation in the wing imaginal disc epithelium but does not occur upon more ubiquitous activation [25].

Interestingly, although expressing one copy of CagA^EPISA^ with the bx-GAL4 driver did not cause a phenotype (Figure 1F), expressing two copies induced formation of small apoptotic clusters within the expression domain (Figure 1G). This reduction in apoptosis induction suggests that the phenomenon does not require phosphorylated CagA, but that CagA^EPISA^ is a less potent activator of cell death. This observation is consistent with data obtained from transgenic expression of CagA^EPISA^ in the eye imaginal disc epithelium, where less severe phenotypes were shown to result from differential cellular localization of the phosphorylation-resistant form of CagA. Whereas wild type CagA was highly enriched at the apical membrane in eye imaginal disc epithelial cells, CagA^EPISA^ was expressed diffusely throughout the cytoplasm. We propose that the inability of phosphorylation-resistant CagA to localize apically within an epithelium influences its interactions with host cell proteins and their resulting effects on the epithelial tissue [9].

Cells within the apoptotic clusters generated by CagA expression were extruded from the basal surface of the wing imaginal disc epithelium. Further examination of this tissue revealed an enrichment of matrix metalloproteinases, which break down basement membrane, specifically in cells located directly apical to the apoptotic clusters (Figure 1H). This observation indicates that apoptotic cells generated by CagA expression are actively removed from the wing epithelium and not passively lost during development of the imaginal disc.

Many complex cellular interactions are required during wing disc development to ensure proper formation of the adult wing structure (Figure 1I). While this process did not appear to be disrupted by ubiquitous expression of CagA in the wing (Figure 1J), CagA expression specifically in the dorsal wing caused a dose-dependent disruption of the imaginal disc epithelium (Figure S1M and S1N) which affected the overall appearance of the adult wing (Figure 1K and 1L). This phenomenon also did not require phosphorylated CagA since expression of CagA^EPISA^ caused a less severe dose-dependent disruption of the adult wing (Figure 1M and 1N). The observation that ubiquitous expression of CagA in the wing does not cause apoptosis or epithelial disruption suggests that wild type cells surrounding those which express CagA are required to produce both phenotypes. This is consistent with the previous observation that JNK-dependent apoptosis is only triggered when aberrant cells within an epithelium are surrounded by wild type cells [26]. Taken together, these data prompted us to examine a potential role for JNK signaling in the apoptosis and epithelial disruption phenotypes resulting from localized expression of CagA in the wing imaginal disc.

### CagA-induced apoptosis occurs through activation of the JNK signaling pathway

**Figure 2:**
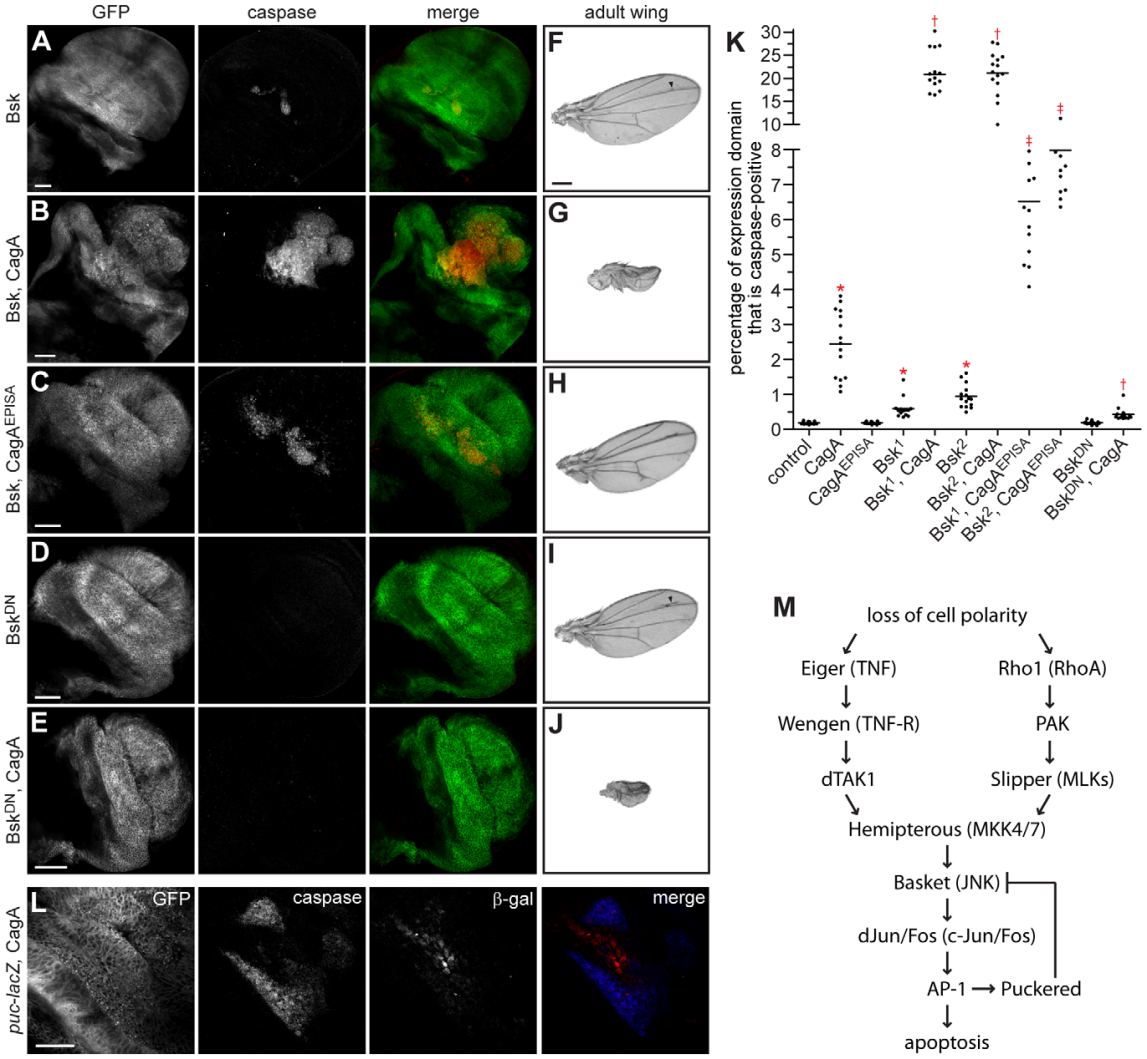
CagA-induced apoptosis occurs through JNK pathway activation. (A–E) Confocal cross sections of male third instar larval wing imaginal discs showing mGFP expression with bx-GAL4 and stained with an anti-active caspase-3 antibody to mark apoptotic cells. Ectopic overexpression of wild type Bsk in the dorsal wing disc (A) causes a mild apoptosis phenotype that is strongly enhanced by coexpression with CagA (B). Coexpression of Bsk with CagA^EPISA^ (C) also enhances the apoptosis phenotype. Expression of Bsk^DN^ alone (D) does not cause apoptosis, and coexpression with CagA (E) strongly suppresses apoptosis induced by CagA expression. Scale bars, 50 µm. (F–J) Adult wing images from male flies expressing different forms of Bsk alone or in combination with CagA. Ectopic overexpression of Bsk with bx-GAL4 (F) causes only subtle vein defects in the adult wing, while coexpression with CagA (G) enhances epithelial disruption. Coexpression of Bsk with CagA^EPISA^ (H) does not significantly affect formation of the adult wing structure. Expression of Bsk^DN^ with bx-GAL4 (I) also causes only subtle vein defects in the adult wing, while coexpression with CagA (J) enhances epithelial disruption. Arrowheads highlight ectopic veins in adult wings expressing different forms of Bsk alone. Scale bar, 500 µm. (K) Quantitation of apoptosis as a percentage of the expression domain showing active caspase-3 staining, n = 15 wing discs per genotype; bar indicates average value for each group. * indicates values that differ significantly from the control with expression of a single transgene; † indicates values that show significant enhancement or suppression compared to CagA; ‡ indicates values that show significant enhancement compared to CagA^EPISA^; p<0.0001. (L) Confocal cross section of a male wing imaginal disc epithelium carrying the *puc-lacZ* reporter allele and expressing mGFP and CagA with bx-GAL4. Staining with antibodies against active caspase-3 and β-galactosidase (β-gal) shows that apoptotic cells lie adjacent to those in which JNK signaling has been activated. Scale bar, 50 µm. (M) A model of the JNK pathway depicting the multiple upstream activators known to induce JNK-dependent apoptosis in *Drosophila*, and indicating human homologs for each pathway component.

Several aspects of the apoptosis phenotype caused by CagA expression in the wing imaginal disc suggested an interaction between CagA and the JNK pathway. In order to determine the nature of this potential interaction, we examined the effects of expressing several forms of Bsk, the *Drosophila* homolog of JNK, on the CagA-induced wing phenotype. Ectopic overexpression of wild type Bsk with the bx-GAL4 dorsal wing driver generated small apoptotic clusters (Figure 2A), indicating that the presence of excess JNK in the wing can phenocopy CagA expression. Furthermore, the cell death phenotype caused by CagA expression in the wing was dramatically enhanced by coexpression with wild type Bsk (Figure 2B). Coexpression of Bsk with CagA^EPISA^ also caused a substantial amount of apoptosis in the wing imaginal disc, suggesting that this interaction is not dependent on phosphorylated CagA (Figure 2C). As expected, expression of a dominant-negative form of Bsk (Bsk^DN^) alone did not cause apoptosis in the wing imaginal disc (Figure 2D). Significantly, coexpression of Bsk^DN^ with CagA almost completely suppressed the apoptosis phenotype caused by CagA expression (Figure 2E), indicating that blocking JNK signaling suppresses CagA-dependent cell death in the wing. These data suggest that CagA expression triggers wing imaginal disc apoptosis through JNK pathway activation.

We also examined the effects of JNK pathway modulation on the epithelial disruption phenotype caused by CagA expression. Although ectopic overexpression of wild type Bsk with bx-GAL4 caused only a minor adult wing phenotype in the form of extra vein material (Figure 2F), coexpression of Bsk with CagA dramatically enhanced the epithelial disruption phenotype (Figure 2G). Ectopic overexpression of Bsk with CagA^EPISA^ was not sufficient to induce epithelial disruption (Figure 2H). Expression of Bsk^DN^ also gave rise to only subtle vein defects in an otherwise normal adult wing (Figure 2I). Interestingly, Bsk^DN^ expression was not able to rescue but instead enhanced the epithelial disruption caused by CagA expression (Figure 2J). One explanation for this apparent contradiction is that blocking JNK signaling prevents the induction of apoptosis that is required to remove aberrant CagA-expressing cells from within the epithelium, which are then allowed to accumulate and lead to a more severe disruption of the adult structure. We tested this hypothesis using the apoptosis inhibitor p35, a baculovirus-derived suicide substrate for effector caspases. Overexpressing p35 alone with bx-GAL4 did not produce a phenotype (Figure S2A and S2C), while coexpressing p35 with CagA effectively blocked apoptosis but enhanced disruption of the adult wing epithelium (Figure S2B and S2D). This observation is consistent with the inhibition of apoptosis resulting in more severe CagA-dependent adult phenotypes. Enhancement and suppression of CagA-induced apoptosis in the wing imaginal disc was quantified using a method we developed to measure the percentage of the expression domain that is caspase-positive. These quantitative data showed that both the enhancement of CagA-induced apoptosis seen with coexpression of ectopic Bsk, and its suppression upon expression of Bsk^DN^ were statistically significant (Figure 2K).

In order to further examine the genetic interaction between CagA and JNK signaling, we used a *lacZ* reporter allele of *puckered* (*puc*), the main component of a negative feedback loop in the JNK pathway. This construct has been used extensively as a readout for JNK pathway activation in *Drosophila* tissue using antibody staining for β-galactosidase (β-gal). Expressing CagA in combination with *puc-lacZ* in the dorsal wing imaginal disc demonstrated that cells adjacent to those undergoing apoptosis are activating JNK signaling (Figure 2L). Upregulation of *puc-lacZ* correlated with phosphorylation of JNK, verifying that specific activation of JNK signaling results from CagA expression (Figure S2E). These data provide additional evidence that CagA expression activates JNK signaling in the wing imaginal disc epithelium.

### Loss of neoplastic tumor suppressors and the TNF homolog Eiger enhances CagA-induced apoptosis

**Figure 3:**
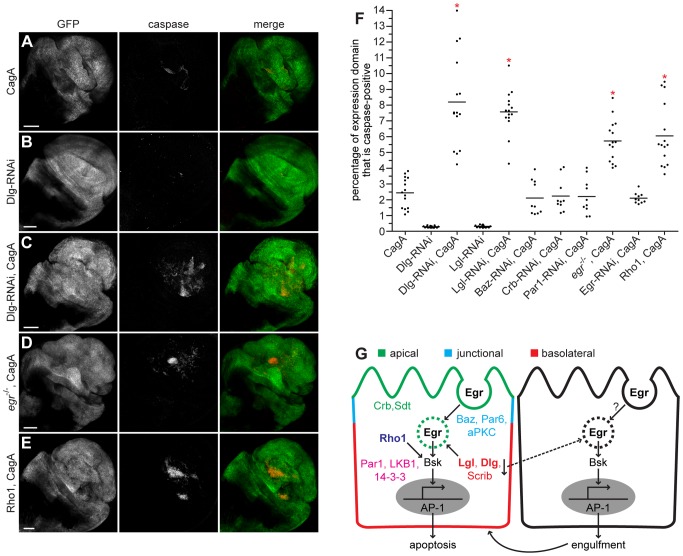
CagA genetically interacts with nTSGs, Eiger and Rho1. (A–E) Confocal cross sections of male third instar larval wing imaginal discs showing mGFP expression with bx-GAL4 and stained with anti-active caspase-3 antibody to mark apoptotic cells. Dorsal wing expression of CagA with bx-GAL4 (A) causes formation of apoptotic clusters. RNAi-mediated knockdown of the nTSG Dlg alone (B) does not cause significant apoptosis, but enhances apoptosis induced by CagA expression (C). The apoptosis phenotype is enhanced when CagA is expressed in an *egr* mutant background (D). Coexpression of Rho1 with CagA (E) also enhances apoptosis. Scale bars, 50 µm. (F) Quantitation of apoptosis as a percentage of the expression domain showing active caspase-3 staining, n = 10 or 15 wing discs per genotype; bar indicates average value for each group. * indicates values that show significant enhancement compared to CagA, whose quantitation (from Figure 2) is provided for comparison; p<0.0001. (G) A model showing the localization of polarity protein complexes in an epithelial cell, their known interactions with other upstream activators of JNK signaling in *Drosophila*, and the downstream effects of these interactions.

JNK signaling is activated by a complex set of signals including TNF and loss of epithelial polarity (Figure 2M). To examine the mechanism through which CagA activates JNK signaling, we used the bx-GAL4 driver to express CagA in combination with RNAi-mediated knockdown of known epithelial polarity determinants and examined wing imaginal discs for enhancement of the apoptosis phenotype (Figure 3A). We tested a panel of polarity proteins, many of which caused apoptosis when knocked down in the absence of CagA expression (Table S1). We chose to target a protein from each of the previously described complexes whose localization and function establish epithelial cell polarity [27], and to simplify our analysis we selected polarity proteins that did not cause an apoptosis phenotype when knocked down on their own (Figure S3A–S3C). When tested in combination with CagA expression, we found that RNAi-mediated knockdown of neither the junctional protein Bazooka (Baz), nor the apical protein Crumbs (Crb) enhanced apoptosis (Figure S3D and S3E). In addition, knockdown of Par1, which has been shown to interact with CagA in tissue culture cells [28], did not enhance the apoptosis phenotype caused by CagA expression in this context (Figure S3F).

Interestingly, RNAi-mediated knockdown of the basolateral protein Discs Large (Dlg) did not cause a significant phenotype (Figure 3B) but markedly enhanced the apoptosis caused by CagA expression (Figure 3C). The same effect was seen with knockdown of Lethal Giant Larvae (Lgl), another basolateral protein (Figure S3G and S3H). The genes encoding these polarity proteins are known as neoplastic tumor suppressor genes (nTSGs) because their loss causes tumor formation in *Drosophila* [29], and generating clones of cells which lack this specific class of polarity determinants has been shown to trigger JNK-dependent apoptosis in imaginal discs [30]. Our data suggest that nTSGs normally suppress CagA-mediated JNK pathway activation and subsequent apoptosis in the wing imaginal disc.

Disruption of the nTSGs activates JNK signaling through endocytosis of the TNF homolog Egr [31]. Homozygous *egr* mutant animals are viable and, as expected, no apoptosis was observed in their wing imaginal discs (Figure S3I). Conversely, ectopic overexpression of wild type Egr in the dorsal wing imaginal disc caused a severe apoptosis phenotype (Figure S3J), consistent with previous data showing Egr to be a potent activator of cell death in *Drosophila* epithelia [32]. We made the unexpected observation that expression of CagA in the dorsal wing disc of an *egr* mutant animal enhanced the apoptosis phenotype (Figure 3D). Interestingly, RNAi-mediated knockdown of Egr alone in the dorsal wing with bx-GAL4 did not cause a phenotype (Figure S3K) or enhance apoptosis when coexpressed with CagA (Figure S3L). This observation suggests that loss of Egr in wild type cells surrounding the CagA expression domain is responsible for the enhanced apoptosis phenotype seen in the wing imaginal discs of *egr* mutant animals expressing CagA.

Recent data has demonstrated that loss of nTSGs in clones of imaginal disc cells causes Egr-dependent activation of nonapoptotic JNK signaling in their wild type neighbors. JNK activation in surrounding wild type cells leads to induction of a phagocytic pathway which triggers engulfment of polarity-deficient cells within the clone [30]. A similar mechanism can be invoked to explain the enhancement of CagA-induced apoptosis seen in *egr* mutant wing imaginal discs. Loss of Egr in the wild type cells surrounding the expression domain may prevent engulfment of CagA-expressing cells. This would increase the number of aberrant cells available to undergo apoptosis upon CagA-mediated activation of JNK signaling via another parallel upstream pathway. We hypothesize that multiple cellular consequences of CagA expression can activate JNK signaling combinatorially. Supporting this view, we demonstrated that CagA-induced apoptosis was enhanced by ectopic overexpression with a wild type form of the small GTPase Rho1 (Figure 3E), another upstream activator of the JNK pathway that did not cause a phenotype when overexpressed alone (Figure S3M), and which our group has shown is activated by CagA [9].

Enhancement of CagA-induced apoptosis in the wing imaginal disc was quantified using the previously described method. These data showed significant enhancement of apoptosis with coexpression of CagA and knockdown of nTSGs, ubiquitous loss of Egr or overexpression of Rho1. Knockdown of several other polarity proteins or Egr in CagA-expressing cells did not enhance the apoptosis phenotype (Figure 3F). Overexpression of Rho1, ubiquitous or localized loss of Egr and knockdown of the other polarity proteins alone did not induce significant apoptosis in the wing imaginal disc (Figure S3N). These observations suggest that specific polarity protein complexes within the cell, as well as other upstream activators are responsible for transducing the signals that lead to JNK pathway activation upon CagA expression in the wing imaginal disc (Figure 3G).

### CagA expression enhances the growth and invasion of tumors generated by expression of oncogenic Ras through JNK pathway activation

**Figure 4:**
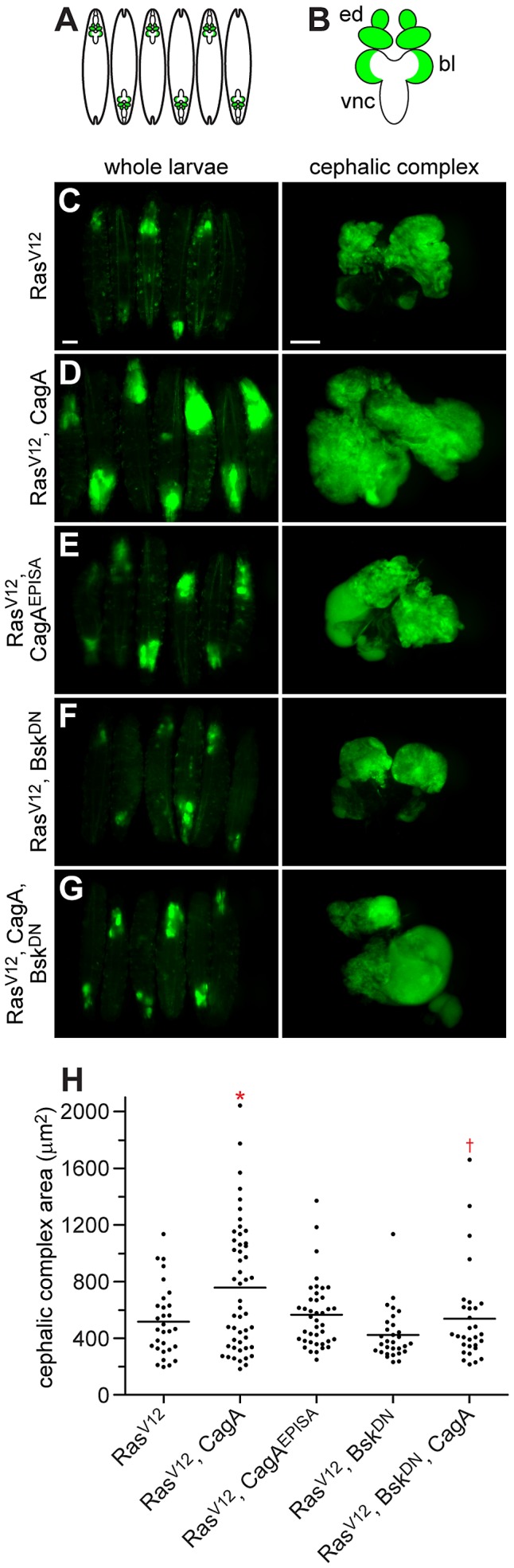
CagA enhances tumor growth through JNK activation. (A–B) Schematics depicting GFP-marked whole eye clones in third instar larvae (A) and a dissected cephalic complex (B), which includes the eye discs (ed), brain lobes (bl) and ventral nerve cord (vnc). (C–G) Images of female third instar larvae and dissected cephalic complexes with GFP-marked tumors. Expression of Ras^V12^ in whole eye clones (C) causes overgrowth which results in tumor formation. Coexpression of CagA with Ras^V12^ (D) markedly enhances the size of tumors, while coexpression of CagA^EPISA^ with Ras^V12^ (E) causes only a minor enhancement of tumor growth. Whole eye clone expression of Bsk^DN^ with Ras^V12^ (F) does not significantly alter tumor size, while coexpression of Bsk^DN^ with Ras^V12^ and CagA (G) suppresses the growth advantage conferred by CagA expression. Scale bar for whole larvae images, 1 mm; scale bar for dissected cephalic complex images, 250 µm. (H) Quantitation of cephalic complex size as a measure of area in µm^2^, n = at least 30 cephalic complexes per genotype; bar indicates average value for each group. * indicates significant enhancement compared to Ras^V12^; † indicates significant suppression compared to Ras^V12^, CagA; p<0.05.

The finding that CagA activates the JNK pathway is intriguing in light of recent evidence indicating that activation of JNK signaling can switch from proapoptotic to progrowth in the presence of oncogenic Ras [19]. In order to examine a potential role for CagA-mediated JNK pathway activation in promoting tumorigenesis, we used a slight variation of a previously established *Drosophila* metastasis model to create whole eye clones expressing an activated form of the Ras oncogene (Ras^V12^) in epithelial cells of the eye imaginal disc using the eyeless (ey) driver with the FLP/FRT system to generate primary tumors [33]. We then evaluated the size of GFP-marked tumors in whole larvae (Figure 4A) and dissected cephalic complexes (Figure 4B) in order to determine whether coexpression of CagA could enhance the growth and invasive potential of these tumor cells through activation of the JNK signaling pathway.

Expression of Ras^V12^ alone in whole eye clones caused overgrowth of eye imaginal disc cells which resulted in tumor formation (Figure 4C), as previously described [34]. Although generating whole eye clones expressing either GFP alone (Figure S4A) or with CagA (Figure S4B) was not tumorigenic, coexpression of CagA enhanced the growth of tumors generated by Ras^V12^ expression (Figure 4D). Whole eye clones expressing CagA^EPISA^ were also not tumorigenic (Figure S4C), and when combined with Ras^V12^ expression caused only a minor enhancement of tumor growth (Figure 4E). As expected, coexpression of Bsk^DN^ did not affect the growth of tumors generated by Ras^V12^ expression alone (Figure 4F). However, Bsk^DN^ expression caused a severe reduction in the growth of tumors expressing both Ras^V12^ and CagA (Figure 4G). Quantification of these data was accomplished by determining the size of dissected cephalic complexes of each genotype and showed a significant growth enhancement with combined expression of Ras^V12^ and CagA, which was suppressed by coexpression of Bsk^DN^ (Figure 4H). These data demonstrate that expression of CagA can enhance the growth of tumors generated by expression of Ras^V12^ in a JNK-dependent manner.

**Figure 5:**
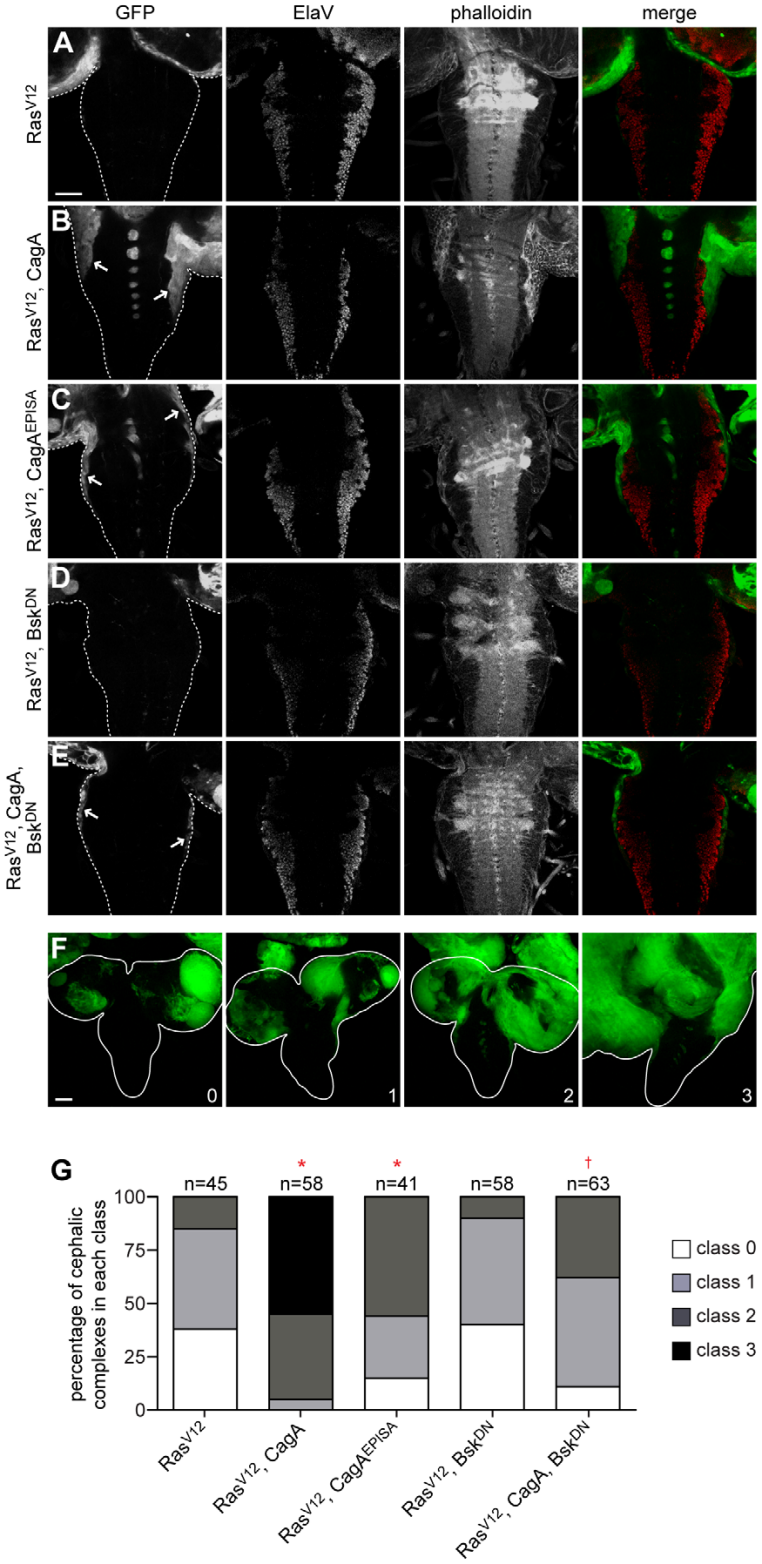
CagA enhances tumor invasion through JNK activation. (A–E) Confocal cross sections of cephalic complexes from third instar larvae with GFP-marked tumors stained with an antibody against ElaV to mark terminally differentiated cells and phalloidin to reveal f-actin structure. VNCs are outlined in panels showing GFP expression, and arrows highlight invading tumor tissue. Expressing Ras^V12^ alone in whole eye clones (A) causes a mild invasive phenotype characterized by either no invasion or migration of tumor cells from one optic lobe. Coexpression of CagA with Ras^V12^ (B) dramatically enhances the extent of VNC invasion from both optic lobes, while coexpression of CagA^EPISA^ with Ras^V12^ (C) shows a milder enhancement of invasion. Coexpression of Bsk^DN^ with Ras^V12^ (D) does not significantly affect the invasive capacity of tumor cells, while coexpression of Bsk^DN^ with Ras^V12^ and CagA (E) suppresses the VNC invasion phenotype. Scale bar, 50 µm. (F) Projections of several confocal cross sections from third instar larval cephalic complexes with GFP-marked tumors showing different classes of invasiveness: (0) noninvasive, (1) invasion from one optic lobe, (2) invasion from both optic lobes, (3) significant invasion of the VNC. Brain lobes and ventral nerve cords are outlined. Scale bar, 50 µm. (G) Quantitation of the percentage of cephalic complexes classified into each category. The number of samples analyzed is shown above each column. * indicates a distribution that differs significantly compared to Ras^V12^; † indicates a distribution that differs significantly compared to Ras^V12^, CagA; p<0.0001.

Generating whole eye clones that express Ras^V12^ alone most commonly caused either a mildly invasive phenotype characterized by the migration of a small number of GFP-positive cells along one edge of the ventral nerve cord (VNC), or a noninvasive phenotype in which cells within the optic lobe approached but did not migrate into the VNC (Figure 5A). Whole eye clones expressing either GFP alone (Figure S5A) or with CagA (Figure S5B) were not invasive, but coexpression of CagA with Ras^V12^ resulted in a much larger number of GFP-positive tumor cells migrating from both optic lobes into the VNC (Figure 5B). These cells were not terminally differentiated, as indicated by a lack of staining with the neuron-specific ElaV antibody, and phalloidin staining showed a morphology distinct from other cells in the VNC (Figure S5D). Expressing CagA^EPISA^ in whole eye clones also did not produce an invasive phenotype (Figure S5C), and coexpression of CagA^EPISA^ with Ras^V12^ caused a less pronounced enhancement of the mild invasion caused by expression of Ras^V12^ alone (Figure 5C), suggesting that the phosphorylation-resistant form of CagA is less effective at promoting tumor progression. Coexpression of Bsk^DN^ did not affect the invasive phenotype generated by Ras^V12^ expression alone (Figure 5D), but Bsk^DN^ expression caused a dramatic reduction in the invasive capacity of tumors expressing both Ras^V12^ and CagA (Figure 5E). These data show that CagA expression can enhance the invasion of Ras^V12^-expressing tumor cells through JNK activation.

In order to determine the significance of CagA's enhancement of invasion, we used a previously described method [35] to categorize invasive phenotypes into four distinct classes which represent a progression from non-invasive to severe invasion of the VNC (Figure 5F). Quantitation of the percentage of cephalic complexes exhibiting each class of VNC invasion showed a significant difference between expression of Ras^V12^ alone and in combination with CagA, which was suppressed by coexpression of Bsk^DN^ (Figure 5G).

## Discussion

**Figure 6:**
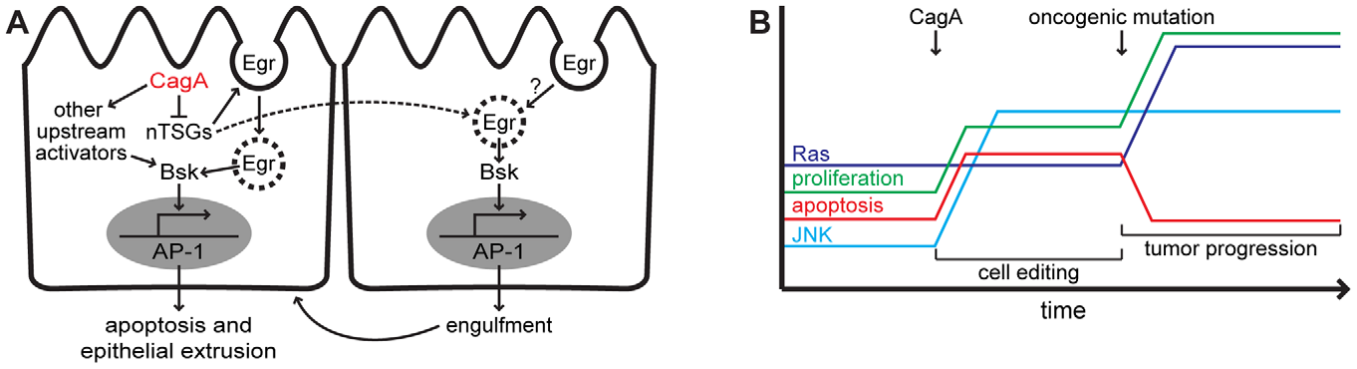
Models illustrating short-term effects of CagA on an epithelium and long-term effects resulting from a change in host genetic background. (A) Once inside the host epithelial cell, CagA effector protein downregulates the neoplastic tumor suppressors (nTSGs) which induces endocytic activation of the TNF homolog Eiger (Egr) leading to activation of JNK (Bsk). CagA also triggers Egr-dependent JNK pathway activation in neighboring wild type cells. In the absence of this pathway, CagA activates JNK signaling through other upstream pathway components including the small GTPase Rho1. In a wild type host genetic background, CagA-mediated JNK pathway activation causes apoptosis and subsequent extrusion from the epithelium, or engulfment by neighboring cells. (B) Introduction of CagA into host cells causes upregulation of JNK signaling which triggers apoptosis and compensatory proliferation within the epithelium as part of the cell editing process. When the host genetic background is perturbed by expression of an oncogenic mutation which blocks apoptosis, CagA-mediated JNK pathway activation drives tumor progression.

In the current study, we used transgenic expression of the CagA virulence factor in *Drosophila* to demonstrate a role for JNK pathway activation in *H. pylori* pathogenesis. When CagA was expressed in a subset of wing imaginal disc cells juxtaposed to non-expressing cells, the epithelium underwent apoptosis and proper formation of the adult wing structure was disrupted. We showed that the apoptosis phenotype occurs through activation of the JNK signaling pathway. CagA-induced apoptosis was enhanced by loss of nTSGs or ectopic expression of the small GTPase Rho1 in the CagA-expressing cells and loss of the TNF homolog Egr in non-expressing cells (Figure 6A). We next showed that CagA-mediated JNK pathway activation can enhance the growth and invasion of tumors generated by expression of oncogenic Ras. Our data uncover a novel genetic interaction between CagA and JNK signaling and demonstrate its potential importance in promoting tumor progression.

### Distribution of CagA within an epithelium can affect manipulation of host proteins and intercellular interactions

Infection of tissue culture cells with *H. pylori* has been shown to activate JNK signaling, but a role for CagA in this process remains controversial [36]–[38]. Additionally, these experiments were performed in nonpolar AGS cells, so if polarity disruption plays a role in JNK pathway activation downstream of CagA, as our data suggest, these cell culture models may not reveal this interaction. JNK pathway activation has also been shown to result from infection with several pathogenic bacteria in epithelial cell culture models of infection [39]. Interestingly, the enteroinvasive bacterium *Shigella flexneri* was shown to activate JNK and upregulate TNFα expression in both infected and adjacent uninfected epithelial cells in culture [40], similar to our data showing that JNK-mediated tissue responses to CagA expression involve a cell-nonautonomous requirement for TNF/Egr. The distribution of *H. pylori* during infection of the gastric epithelium is known to be heterogeneous [2]. We therefore hypothesize that interactions between cells containing CagA protein and uninfected neighboring cells could also be important for pathogenesis of *H. pylori*.

Our data suggest that CagA is an important mediator of JNK pathway activation during *H. pylori* infection, and identify several host proteins involved in this process. We observe genetic interaction between CagA and nTSGs, but not junctional proteins involved in polarity. This is consistent with recent data from tissue culture cells which demonstrated that CagA-positive strains of *H. pylori* specifically disrupt apicobasal polarity in a polarized monolayer prior to affecting the integrity of cellular junctions [11]. Disruption of nTSGs has been shown to cause JNK-dependent apoptosis, and more recent data indicates that elimination of polarity-deficient cells is dependent on their location within the wing imaginal disc due to varying levels of dMyc throughout the tissue [41]. The extent of aberrant cell removal differs significantly with respect to established gradients of Wnt/Wingless, dMyc and Hippo-Salvador-Warts pathway activation that ensure proper development of the wing [42]. We propose that the extent of variation observed upon CagA expression in the wing with different GAL4 drivers is due to spatial variation in these host cell signaling pathways. Our data also suggest that CagA can activate JNK-dependent apoptosis through multiple upstream pathways. The observation that overexpression of Rho1 enhances CagA-dependent apoptosis in the wing imaginal disc epithelium is consistent with previous data from our group demonstrating a role for CagA in activating the Rho pathway to disrupt epithelial patterning [9].

Use of the unique genetic tools available in *Drosophila* has provided important insight into potential interactions between CagA-expressing cells and neighboring wild type cells. Our observation that loss of TNF/Egr in wild type cells surrounding those expressing CagA can enhance apoptosis, presumably by reducing engulfment of CagA-expressing cells, indicates that the genetic state of uninfected cells may also play a role in *H. pylori* pathogenesis. This finding is important with respect to the established function of TNF/Egr-dependent JNK activation in cell competition induced by intrinsic tumor suppression. Our data suggest that the presence of CagA protein induces changes in signaling and morphology which cause an epithelial cell to be outcompeted by its wild type neighbors through a local mechanism that requires TNF/Egr in the neighboring epithelial cells. Interestingly, *Drosophila* immune cells known as hemocytes have also demonstrated the ability to remove polarity-deficient cells from an epithelium through a more global extrinsic tumor suppression mechanism that is TNF/Egr-dependent [43]. Although we have not explored a role for hemocytes in removal of CagA-expressing wing epithelial cells, it is possible that a related mechanism may occur during *H. pylori* infection of the human stomach through immune surveillance mediated by TNF. Although this specific cytokine is an important component of the initial immune response to infection with a pathogen, TNF is also known to promote tumor progression specifically in the context of chronic inflammation or in the presence of activated Ras [43], [44]. We hypothesize that TNF functions to suppress tumor initiation resulting from the presence of CagA protein in gastric epithelial cells through several mechanisms, but that the inflammatory environment created by prolonged infection with *H. pylori* and the emergence of oncogenic mutations over time cause TNF to promote progression of gastric cancer.

### Genetic changes in host cells can alter the downstream effects of CagA signaling during long-term association with *H. pylori*

Since it was first discovered, JNK has been demonstrated to have both pro-tumorigenic and tumor suppressor functions in different cell types and organs. Studies in *Drosophila* have helped shed light on the genetic contexts in which JNK activation functions to promote tumor progression, namely in the presence of oncogenic Ras [45]. Recently, JNK was shown to be required for activated KRas-induced lung tumor formation in mice [46], suggesting a conserved function of JNK activation in cooperating with activated Ras to promote tumorigenesis in mammals. A potential role for JNK pathway activation has also been explored in mammalian gastric cancer. Activation of JNK signaling has been detected in human gastric cancer samples, and mice lacking JNK1 exhibit a decrease in gastric apoptosis and an attenuation of gastric tumor development induced by the chemical carcinogen *N*-methyl-*N*-nitrosourea [47]. A role for *H. pylori* in the context of mammalian gastric cancers induced by cooperation between JNK and Ras signaling has not been explored.

Our finding that CagA expression can induce JNK-dependent apoptosis in a polarized epithelium is interesting with respect to data suggesting that JNK signaling has evolved as a cell editing mechanism to remove aberrant cells from within an epithelium [18]. Activation of JNK signaling could represent a host response aimed at removing cells containing CagA protein from the gastric epithelium. Similarly, *P. aeruginosa*-mediated activation of JNK signaling in the intestinal epithelium of *Drosophila* can trigger epithelial renewal as a host defense mechanism. However, this process can become pathogenic and lead to dramatic overproliferation of intestinal cells in animals harboring oncogenic Ras mutations [21]. In *H. pylori* infection, which can persist for many years before the development of gastric cancer, JNK-mediated apoptosis could be an effective mechanism to limit pathogenic effects on the gastric epithelium. However, this process of tissue editing can also increase cell turnover, contributing to accumulation of genetic mutations in host cells. Our data show that acquisition of an oncogenic mutation in host epithelial cells experiencing CagA-mediated JNK pathway activation can promote tumor progression, suggesting that this potential host defense strategy can become tumorigenic in certain genetic contexts (Figure 6B).

Transgenic expression of CagA was recently found to cause neoplastic transformation in a mouse model, providing evidence for CagA's role as a bacterial oncoprotein in mammals [48]. The low incidence and delayed development of gastrointestinal tumors in these mice was attributed to lower expression of CagA in the surviving animals, as higher expression was assumed to be lethal during embryogenesis. Additionally, secondary mutations were identified in the tumors, but their potential cooperation with host cell signaling pathways activated by CagA expression was not addressed [48]. Infection with CagA-positive *H. pylori* is also known to induce an invasive phenotype in tissue culture cells [49], but potential effects of the oncogenic mutations present in these immortalized cell lines is unknown. Although we did not demonstrate the sufficiency of CagA to induce tumor phenotypes in our *Drosophila* model, our data support a crucial role for CagA in promoting tumor progression in combination with oncogene activation. We believe that using an inducible expression system in *Drosophila* allowed us to bypass the toxicity observed upon CagA expression in mice and cell culture models, thus revealing novel interactions between CagA and host cell proteins with downstream effects on apoptosis and tumorigenesis.

Although half the world's population is thought to be infected with *H. pylori*, a small percentage of those individuals will develop gastric cancer [2]. This observation indicates that, in addition to the presence of the *cag* PAI in more virulent strains, host genetics must also play a crucial role in determining the outcome of *H. pylori* infection. Our results suggest that a change in host genetics during long-term association with *H. pylori* could cause JNK activation to switch from conferring a protective function against CagA-induced cellular changes to enabling tumor progression. Data collected from tissue biopsies indicate that Ras mutation may play a role in the development of gastric cancer in human patients [50], and our data put forward the idea that enhanced tumorigenic potential created by cooperation between JNK pathway activation via the bacterial genetic factor CagA and sporadic activation of Ras in host cells could drive gastric cancer formation in a subset of *H. pylori* infections.

## Materials and Methods

### Fly strains and generation of whole eye clones

The following fly stocks were used: UAS-CagA, UAS-CagA^EPISA^ [7]; bx-GAL4, sd-GAL4, ap-GAL4, en-GAL4, ptc-GAL4, hs-FLP, Act>y^+^>GAL4, UAS-GFP.S65T, UAS-mCD8::GFP (mGFP), UAS-bsk.B (Bsk^1^), UAS-bsk.A-Y (Bsk^2^), UAS-bsk.K53R (Bsk^DN^), *egr* ^MB06803^ (*egr* ^−/−^), UAS-Dlg-RNAi, UASp-FLAG.Rho1 (Rho1), UAS-Ras85D.V12 (Ras^V12^), UAS-Crb-RNAi, UAS-Patj-RNAi, UAS-Cora-RNAi, UAS-Cdc42-RNAi (from Bloomington Stock Center); 765-GAL4 (provided by Ross Cagan, Mount Sinai School of Medicine); *puc* ^E69^ (*puc-lacZ*), Regg1^GS9830^ (UAS-Egr) (provided by Michael Galko, MD Anderson Cancer Center); UAS-Lgl-RNAi, UAS-Baz-RNAi, UAS-Par1-RNAi, UAS-Scrib-RNAi, UAS-Par6-RNAi, UAS-aPKC-RNAi, UAS-Mir-RNAi (provided by Chris Doe, University of Oregon); UAS-Egr-RNAi (from Vienna *Drosophila* Resource Center); ey-FLP; Act>y^+^>GAL4, UAS-GFP (provided by Tory Herman, University of Oregon). Flies were raised at 25°C using standard methods. Whole eye clones were generated as previously described [33] without the GAL80 repressor to express transgenes in all cells that give rise to the eye-antennal disc. FLP-out clones were generated by subjecting each 4–6 hour collection of embryos to one hour of heat-shock at 37°C, then dissecting wing discs approximately 96–120 hours later.

### Histology

Larval tissues were fixed and stained using standard protocols. The following primary antibodies were used: rabbit anti-active caspase-3 (1∶200; BD Pharmingen), mouse anti-Mmp1 (1∶50; Developmental Studies Hybridoma Bank), mouse anti-β-galatosidase (1∶500; Sigma) rat anti-ElaV (1∶10; Developmental Studies Hybridoma Bank), rabbit anti-β-galatosidase (1∶200; MP Biomedicals) and mouse anti-phospho-SAPK/JNK (1∶100; Cell Signaling Technology). Both Cy3 and Cy5-conjugated secondary antibodies were used (1∶200; Jackson ImmunoResearch), as well as Alexa Fluor 546 and Alexa Fluor 633 phalloidin (1∶40; Molecular Probes). Intact adult wings were mounted in a 1∶1 mixture of lactic acid and ethanol.

### Image analysis and quantitation

Adult wings, intact larvae and whole cephalic complexes were visualized using light microscopy or GFP fluorescence on a Zeiss dissecting microscope. Wing imaginal discs, ventral nerve cords and cephalic complexes were visualized on a Nikon confocal microscope. Images were processed using Adobe Photoshop, where levels were adjusted to optimize the signal-to-noise ratio in each color channel while maintaining similar levels of background noise and desired signal between channels and images. Adult wing images were removed from their background using the Extract filter in Adobe Photoshop. XZ confocal planes were created using the Reslice function in Image J. Projections of confocal cross sections were created using the Merge to HDR command in Adobe Photoshop. Apoptosis was quantified by selecting the single confocal cross section of each wing imaginal disc exhibiting the highest level of active caspase-3 staining and manually tracing the expression domain, then determining the percentage of this domain showing active caspase-3 staining using the Threshold function in Image J. Cephalic complex size was quantified using the Threshold function in Image J to determine the area of the tissue in µm^2^. Graphs were created with GraphPad Prism software, which was also used to calculate two-tailed p values using the unpaired t test with Welch's correction for apoptosis quantitation. The statistical significance of differences in metastatic potential for each genotype was calculated using Excel to determine two-tailed p values using the unpaired t test.

## Supporting Information

Figure S1 **The effects of CagA depend on its expression pattern in the wing, and CagA expression in the dorsal wing imaginal disc disrupts epithelial integrity.** (A) Schematic illustrating expression domains of the various GAL4 drivers used to express CagA in the wing imaginal disc. (B–K) Confocal cross sections of third instar larval wing imaginal discs showing GFP expression, and stained with an antibody against active caspase-3 to mark apoptotic cells and phalloidin to reveal f-actin structure. Generating clones of wing imaginal disc cells expressing GFP alone (B) or in combination with CagA (C) does not cause any observable phenotype. Expressing mGFP alone with the scalloped-GAL4 driver (sd) does not cause a phenotype (D), but expressing CagA induces apoptosis in the wing blade region of the imaginal disc (E). Using the apterous-GAL4 driver (ap) to express mGFP alone does not cause a phenotype (F), but expression of CagA triggers apoptosis in the dorsal wing blade region of the imaginal disc (G). Expressing mGFP alone with the engrailed-GAL4 driver (en) does not cause a phenotype (H), but expressing CagA causes disruption of the imaginal disc epithelium (I). Using the patched-GAL4 driver (ptc) to express mGFP alone (J) does not cause a phenotype, but expression of CagA triggers slight epithelial disruption and very mild apoptosis in the wing blade region of the imaginal disc (K). Scale bar, 50 µm. (L) Adult wing images from male flies expressing mGFP and CagA with the indicated GAL4 driver, which show varying amounts of epithelial disruption. Scale bar, 500 µm. (M–N) Confocal cross sections of third instar larval wing imaginal discs showing mGFP expression, and stained with an antibody against active caspase-3 to mark apoptotic cells and phalloidin to reveal f-actin structure. Expressing CagA with bx-GAL4 disrupts normal epithelial architecture most significantly in regions of the wing imaginal disc that are undergoing apoptosis (M). Epithelial disruption is more significant in wing imaginal discs expressing two copies of CagA with bx-GAL4, which exhibit this phenotype throughout the tissue (N). Scale bar, 50 µm.
(TIF)

Figure S2 **Apoptosis inhibition enhances CagA-dependent epithelial disruption, and the puc-lacZ reporter allele functions as a specific readout of CagA-mediated JNK pathway activation.** (A–B) Confocal cross sections of male third instar larval wing imaginal discs showing mGFP expression with bx-GAL4 and stained with anti-active caspase-3 antibody to mark apoptotic cells. Ectopic overexpression of p35 in the dorsal wing disc does not cause a phenotype (A), and coexpression with CagA suppresses the apoptosis normally caused by CagA expression (B). Scale bars, 50 µm. (C–D) Adult wing images from male flies expressing the apoptosis inhibitor p35 alone or in combination with CagA. Ectopic expression of p35 with bx-GAL4 does not cause a phenotype (C), while coexpression with CagA enhances epithelial disruption (D). Scale bar, 500 µm. (E) Confocal cross section of a male wing imaginal disc epithelium carrying the *puc-lacZ* reporter allele and expressing mGFP and CagA with bx-GAL4. Staining with antibodies against β-galactosidase (β-gal) and phosphorylated JNK (p-JNK) shows that *puc-lacZ* upregulation correlates with JNK phosphorylation. Scale bar, 50 µm.
(TIF)

Figure S3 **Manipulation of specific polarity determinants and upstream activators of JNK signaling enhances CagA-induced apoptosis**. (A–M) Confocal cross sections of male third instar larval wing imaginal discs showing mGFP expression with bx-GAL4 and stained with anti-active caspase-3 antibody to mark apoptotic cells. RNAi-mediated knockdown of polarity determinants Baz (A), Crb (B) or Par1 (C) alone in the dorsal wing does not induce apoptosis. Coexpression of CagA with knockdown of Baz (D), Crb (E) or Par1 (F) does not enhance the apoptosis phenotype. Knockdown of the neoplastic tumor suppressor Lgl alone also does not cause significant apoptosis (G), but when combined with CagA expression markedly enhances apoptosis (H). Wing imaginal discs of *egr* mutant animals do not exhibit apoptosis (I). Ectopic expression of Egr alone in the dorsal wing causes a significant apoptosis phenotype (J). RNAi-mediated knockdown of Egr alone does not cause apoptosis (K), and does not enhance the apoptosis phenotype when combined with CagA expression (L). Ectopic expression in the dorsal wing of the small GTPase Rho1 alone does not cause apoptosis (M). Scale bars, 50 µm. (N) Quantitation of apoptosis as a percentage of the expression domain showing active caspase-3 staining, n = 5 wing discs per genotype; bar indicates average value for each group. None of these values show significant apoptosis compared to the control, whose quantitation (from Figure 2) is provided for comparison.
(TIF)

Figure S4 **Expression of CagA alone does not induce cephalic complex overgrowth.** (A–C) Images of dissected cephalic complexes expressing GFP with ey-GAL4. Expression of GFP alone (A), with CagA (B) or with CagA^EPISA^ (C) in whole eye clones does not cause overgrowth or result in tumor formation. Scale bar, 250 µm.
(TIF)

Figure S5 **Expression of CagA alone does not induce ventral nerve cord invasion, and invasive tumor tissue is morphologically distinct from other cells in the ventral nerve cord.** (A–D) Confocal cross sections of cephalic complexes from third instar larvae expressing GFP with ey-GAL4 and stained with an antibody against ElaV to mark terminally differentiated cells and phalloidin to reveal f-actin structure. In panels showing GFP expression, VNCs are outlined. Expression of GFP alone (A), with CagA (B) or with CagA^EPISA^ (C) in whole eye clones does not cause an invasive phenotype. Scale bar, 50 µm. (D) Invasive tumor tissue is morphologically distinct from other cells in the VNC, as visualized by magnification of the metastatic region of the cephalic complex shown in Figure 5B.
(TIF)

Table S1 **Knockdown of specific polarity determinants in the wing causes apoptosis and epithelial disruption, and enhances CagA-dependent phenotypes.** Expression of each polarity determinant was subject to RNAi-mediated knockdown using the bx-GAL4 driver. Effects on both apoptosis in the wing imaginal disc and epithelial disruption in the adult wing were determined. Those proteins whose knockdown alone did not produce a significant phenotype were tested for their ability to enhance or suppress CagA-dependent phenotypes in the larval and adult wing.
(DOCX)
