# Supplementary figures and images for: Transgenic Expression of the Helicobacter pylori Virulence Factor CagA Promotes Apoptosis or Tumorigenesis through JNK Activation in Drosophila
Source: PLoS Pathog. 2012 Oct 18;8(10):e1002939. doi: 10.1371/journal.ppat.1002939 (PMC3475654; doi:10.1371/journal.ppat.1002939)

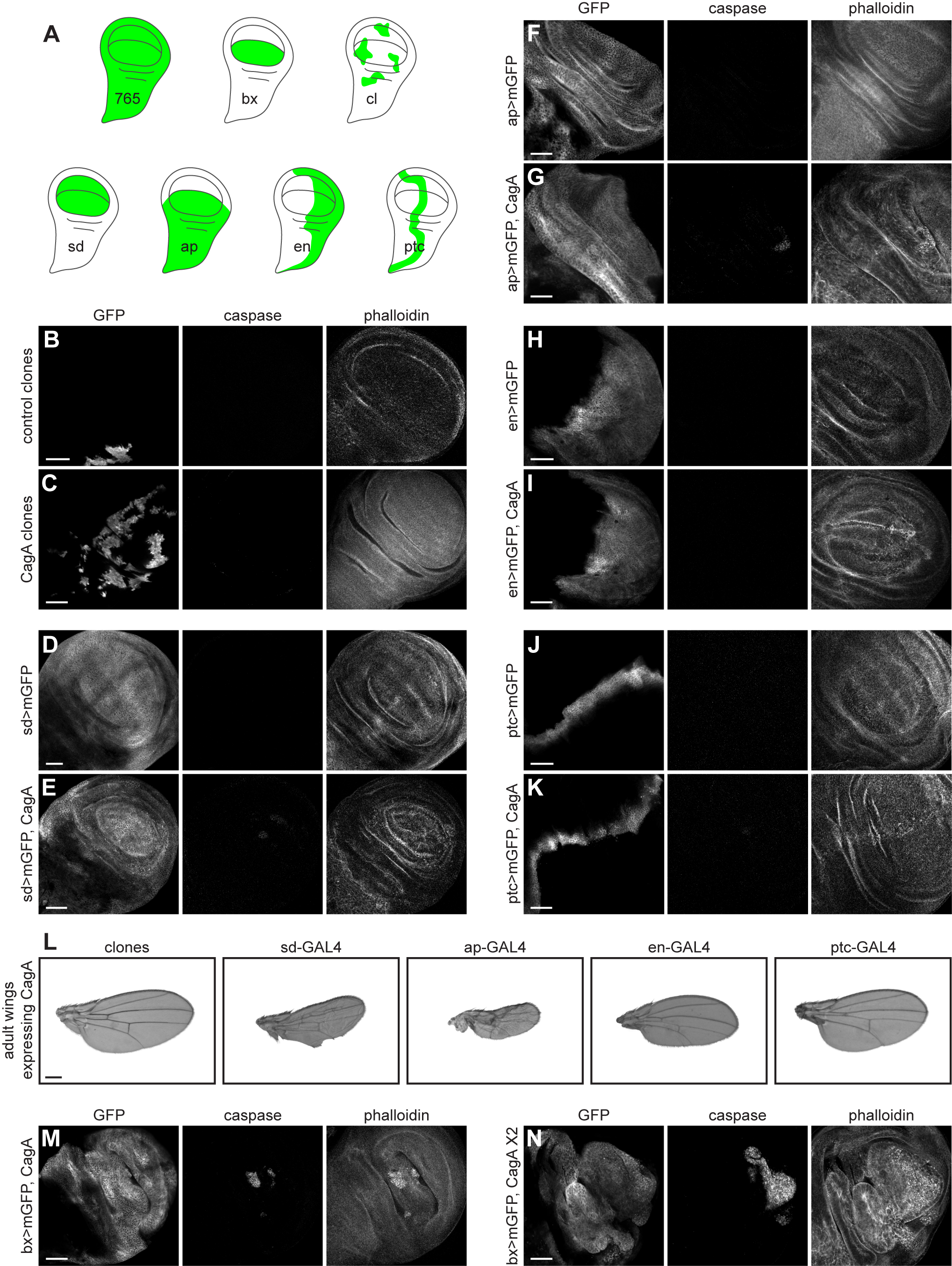

Supplement: Figure S1 — The effects of CagA depend on its expression pattern in the wing, and CagA expression in the dorsal wing imaginal disc disrupts epithelial integrity. (A) Schematic illustrating expression domains of the various GAL4 drivers used to express CagA in the wing imaginal disc. (B–K) Confocal cross sections of third instar larval wing imaginal discs showing GFP expression, and stained with an antibody against active caspase-3 to mark apoptotic cells and phalloidin to reveal f-actin structure. Generating clones of wing imaginal disc cells expressing GFP alone (B) or in combination with CagA (C) does not cause any observable phenotype. Expressing mGFP alone with the scalloped-GAL4 driver (sd) does not cause a phenotype (D), but expressing CagA induces apoptosis in the wing blade region of the imaginal disc (E). Using the apterous-GAL4 driver (ap) to express mGFP alone does not cause a phenotype (F), but expression of CagA triggers apoptosis in the dorsal wing blade region of the imaginal disc (G). Expressing mGFP alone with the engrailed-GAL4 driver (en) does not cause a phenotype (H), but expressing CagA causes disruption of the imaginal disc epithelium (I). Using the patched-GAL4 driver (ptc) to express mGFP alone (J) does not cause a phenotype, but expression of CagA triggers slight epithelial disruption and very mild apoptosis in the wing blade region of the imaginal disc (K). Scale bar, 50 µm. (L) Adult wing images from male flies expressing mGFP and CagA with the indicated GAL4 driver, which show varying amounts of epithelial disruption. Scale bar, 500 µm. (M–N) Confocal cross sections of third instar larval wing imaginal discs showing mGFP expression, and stained with an antibody against active caspase-3 to mark apoptotic cells and phalloidin to reveal f-actin structure. Expressing CagA with bx-GAL4 disrupts normal epithelial architecture most significantly in regions of the wing imaginal disc that are undergoing apoptosis (M). Epithelial disruption is more significan [file ppat.1002939.s001.tif]

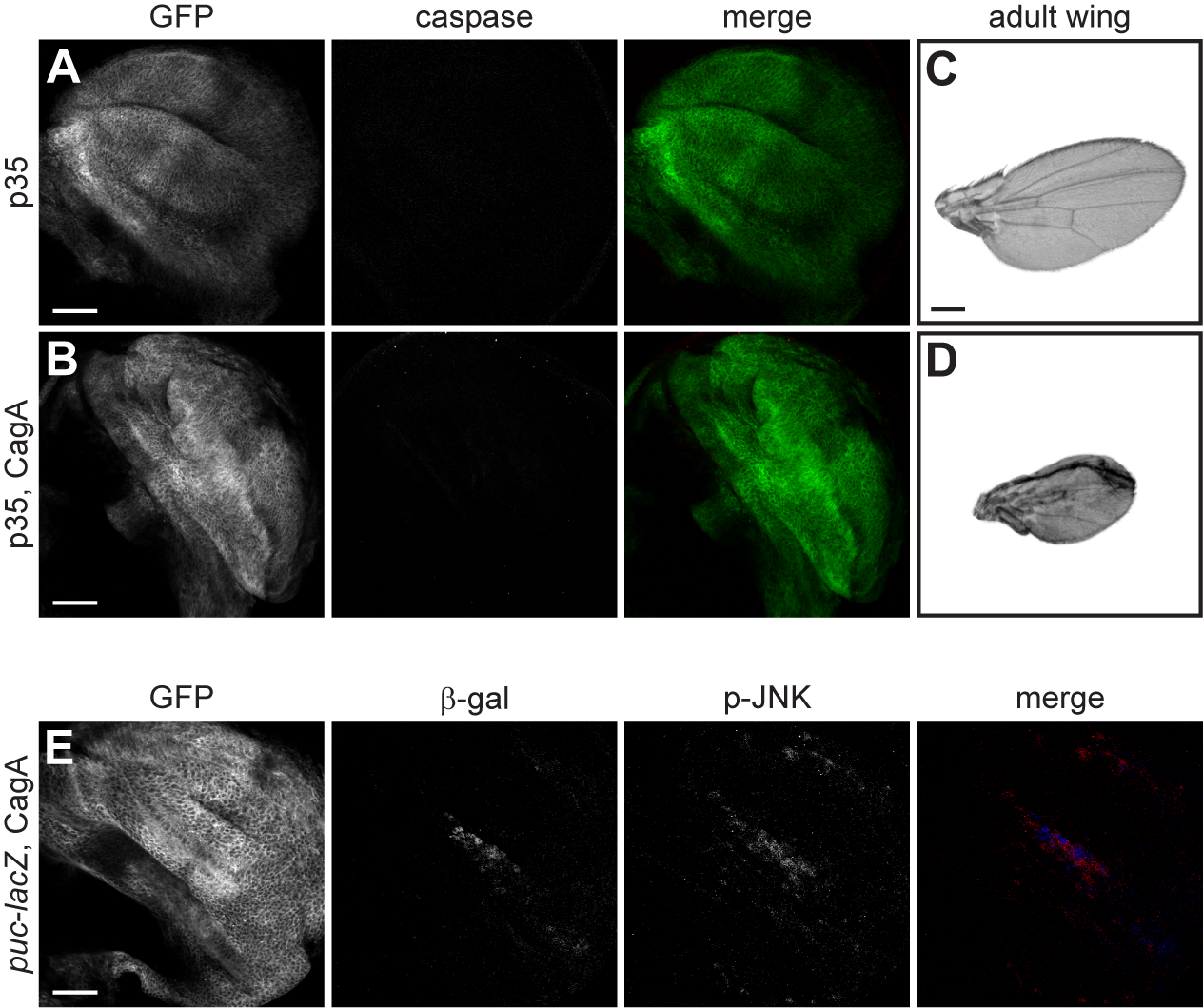

Supplement: Figure S2 — Apoptosis inhibition enhances CagA-dependent epithelial disruption, and the puc-lacZ reporter allele functions as a specific readout of CagA-mediated JNK pathway activation. (A–B) Confocal cross sections of male third instar larval wing imaginal discs showing mGFP expression with bx-GAL4 and stained with anti-active caspase-3 antibody to mark apoptotic cells. Ectopic overexpression of p35 in the dorsal wing disc does not cause a phenotype (A), and coexpression with CagA suppresses the apoptosis normally caused by CagA expression (B). Scale bars, 50 µm. (C–D) Adult wing images from male flies expressing the apoptosis inhibitor p35 alone or in combination with CagA. Ectopic expression of p35 with bx-GAL4 does not cause a phenotype (C), while coexpression with CagA enhances epithelial disruption (D). Scale bar, 500 µm. (E) Confocal cross section of a male wing imaginal disc epithelium carrying the puc-lacZ reporter allele and expressing mGFP and CagA with bx-GAL4. Staining with antibodies against β-galactosidase (β-gal) and phosphorylated JNK (p-JNK) shows that puc-lacZ upregulation correlates with JNK phosphorylation. Scale bar, 50 µm. (TIF) [file ppat.1002939.s002.tif]

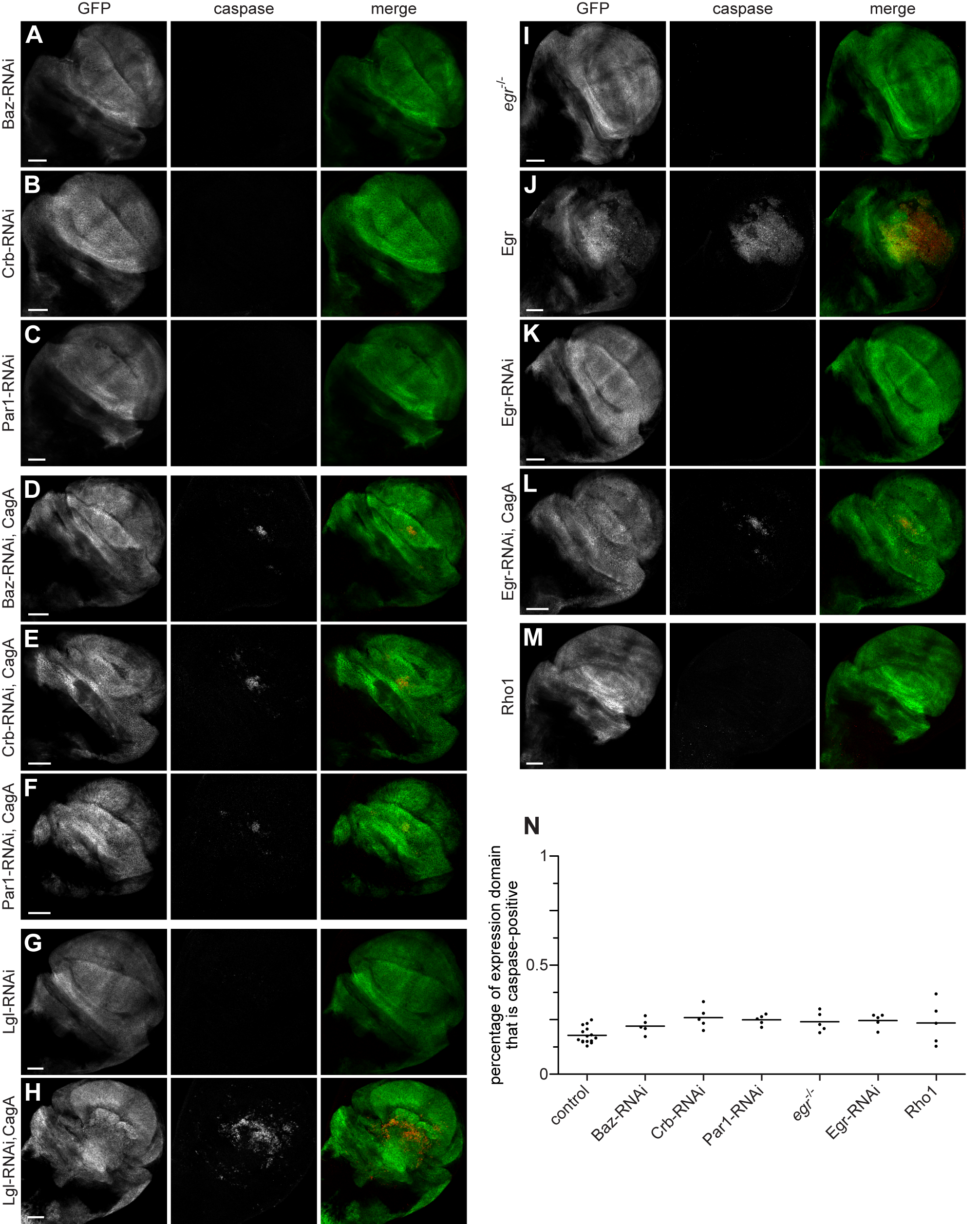

Supplement: Figure S3 — Manipulation of specific polarity determinants and upstream activators of JNK signaling enhances CagA-induced apoptosis. (A–M) Confocal cross sections of male third instar larval wing imaginal discs showing mGFP expression with bx-GAL4 and stained with anti-active caspase-3 antibody to mark apoptotic cells. RNAi-mediated knockdown of polarity determinants Baz (A), Crb (B) or Par1 (C) alone in the dorsal wing does not induce apoptosis. Coexpression of CagA with knockdown of Baz (D), Crb (E) or Par1 (F) does not enhance the apoptosis phenotype. Knockdown of the neoplastic tumor suppressor Lgl alone also does not cause significant apoptosis (G), but when combined with CagA expression markedly enhances apoptosis (H). Wing imaginal discs of egr mutant animals do not exhibit apoptosis (I). Ectopic expression of Egr alone in the dorsal wing causes a significant apoptosis phenotype (J). RNAi-mediated knockdown of Egr alone does not cause apoptosis (K), and does not enhance the apoptosis phenotype when combined with CagA expression (L). Ectopic expression in the dorsal wing of the small GTPase Rho1 alone does not cause apoptosis (M). Scale bars, 50 µm. (N) Quantitation of apoptosis as a percentage of the expression domain showing active caspase-3 staining, n = 5 wing discs per genotype; bar indicates average value for each group. None of these values show significant apoptosis compared to the control, whose quantitation (from Figure 2) is provided for comparison. (TIF) [file ppat.1002939.s003.tif]

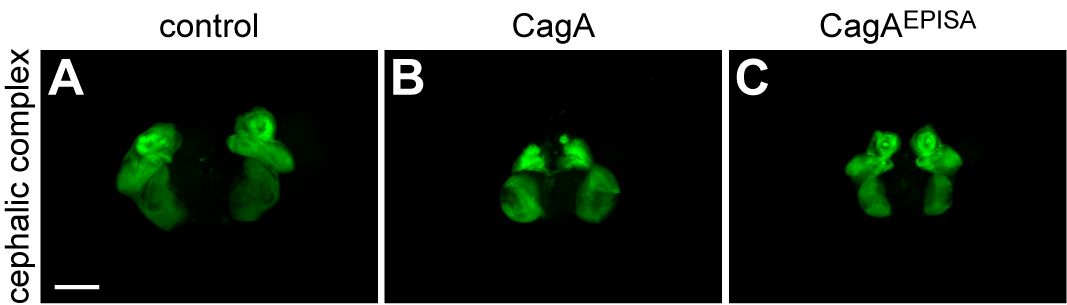

Supplement: Figure S4 — Expression of CagA alone does not induce cephalic complex overgrowth. (A–C) Images of dissected cephalic complexes expressing GFP with ey-GAL4. Expression of GFP alone (A), with CagA (B) or with CagAEPISA (C) in whole eye clones does not cause overgrowth or result in tumor formation. Scale bar, 250 µm. (TIF) [file ppat.1002939.s004.tif]

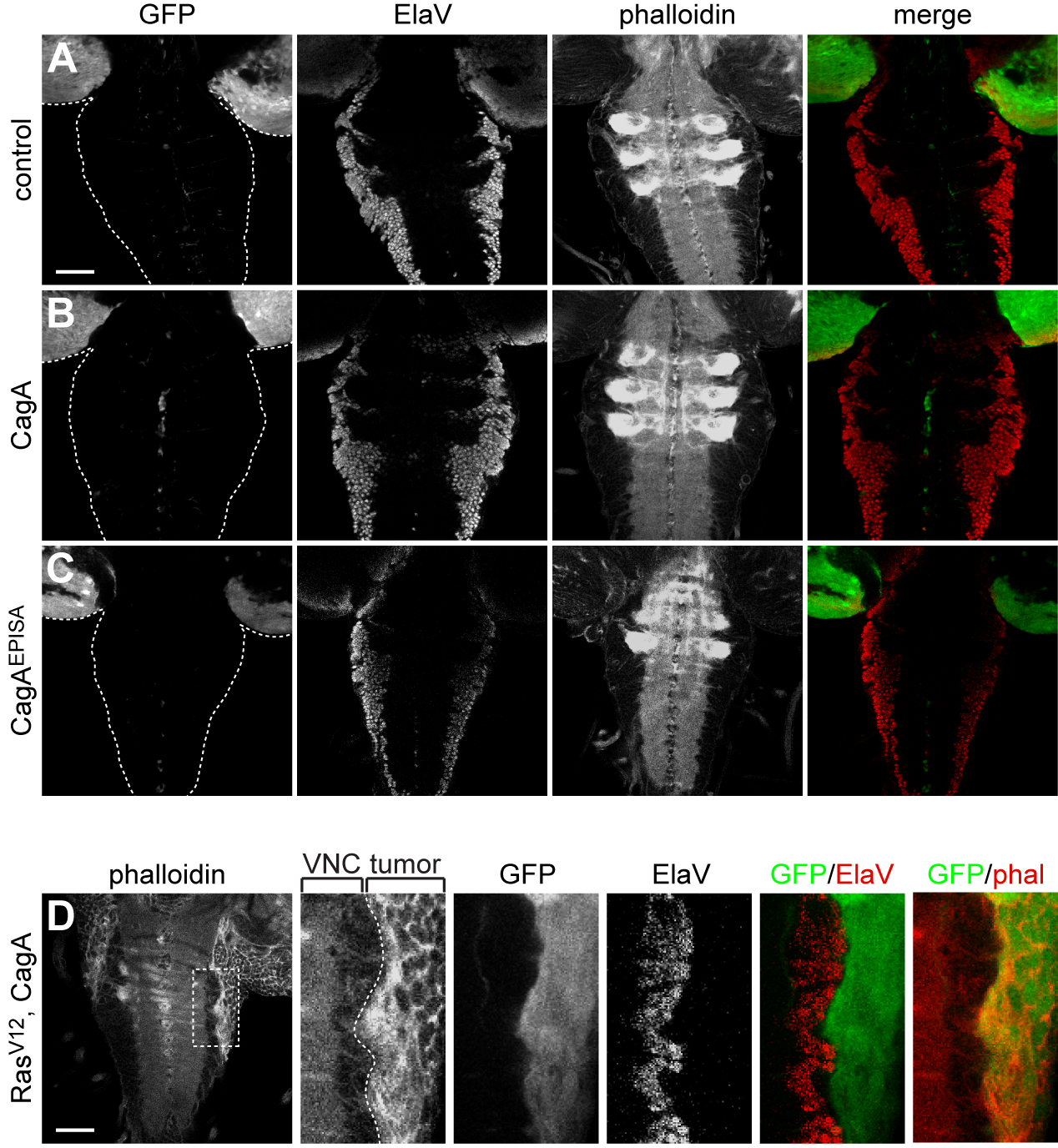

Supplement: Figure S5 — Expression of CagA alone does not induce ventral nerve cord invasion, and invasive tumor tissue is morphologically distinct from other cells in the ventral nerve cord. (A–D) Confocal cross sections of cephalic complexes from third instar larvae expressing GFP with ey-GAL4 and stained with an antibody against ElaV to mark terminally differentiated cells and phalloidin to reveal f-actin structure. In panels showing GFP expression, VNCs are outlined. Expression of GFP alone (A), with CagA (B) or with CagAEPISA (C) in whole eye clones does not cause an invasive phenotype. Scale bar, 50 µm. (D) Invasive tumor tissue is morphologically distinct from other cells in the VNC, as visualized by magnification of the metastatic region of the cephalic complex shown in Figure 5B. (TIF) [file ppat.1002939.s005.tif]
