# Supplementary material for: Transgenic Expression of the Helicobacter pylori Virulence Factor CagA Promotes Apoptosis or Tumorigenesis through JNK Activation in Drosophila
Source: PLoS Pathog. 2012 Oct 18;8(10):e1002939. doi: 10.1371/journal.ppat.1002939 (PMC3475654; doi:10.1371/journal.ppat.1002939)
Supplement: Table S1 — Knockdown of specific polarity determinants in the wing causes apoptosis and epithelial disruption, and enhances CagA-dependent phenotypes. Expression of each polarity determinant was subject to RNAi-mediated knockdown using the bx-GAL4 driver. Effects on both apoptosis in the wing imaginal disc and epithelial disruption in the adult wing were determined. Those proteins whose knockdown alone did not produce a significant phenotype were tested for their ability to enhance or suppress CagA-dependent phenotypes in the larval and adult wing. (DOCX) [file ppat.1002939.s006.docx]

|  | control | |  | CagA | |
| --- | --- | --- | --- | --- | --- |
|  | **apoptosis** | **epithelial disruption** |  | **apoptosis** | **epithelial disruption** |
| **Scrib-RNAi** | severe | severe |  | n/a | n/a |
| **Dlg-RNAi** | none | mild |  | enhanced | enhanced |
| **Lgl-RNAi** | none | mild |  | enhanced | enhanced |
|  |  |  |  |  |  |
| **Crb-RNAi** | none | mild |  | not enhanced | not enhanced |
| **Patj-RNAi** | none | mild |  | not enhanced | enhanced |
| **Cora-RNAi** | moderate | mild |  | n/a | n/a |
| **Par1-RNAi** | none | none |  | not enhanced | not enhanced |
|  |  |  |  |  |  |
| **Baz-RNAi** | none | none |  | not enhanced | enhanced |
| **Par6-RNAi** | severe | severe |  | n/a | n/a |
| **aPKC-RNAi** | severe | severe |  | n/a | n/a |
| **Cdc42-RNAi** | severe | severe |  | n/a | n/a |
|  |  |  |  |  |  |
| **Mir-RNAi** | none | none |  | not enhanced | enhanced |
